# Crystal structure of LRG1 and the functional significance of LRG1 glycan for LPHN2 activation

**DOI:** 10.1038/s12276-023-00992-4

**Published:** 2023-05-01

**Authors:** Jimin Yang, Guo Nan Yin, Do-Kyun Kim, Ah-reum Han, Dong Sun Lee, Kwang Wook Min, Yaoyao Fu, Jeongwon Yun, Jun-Kyu Suh, Ji-Kan Ryu, Ho Min Kim

**Affiliations:** 1 Center for Biomolecular and Cellular Structure, Institute for Basic Science (IBS), Daejeon, 34126 Republic of Korea; 2 National Research Center for Sexual Medicine and Department of Urology, Inha University School of Medicine, Incheon, 22332 Republic of Korea; 3 Korea Zoonosis Research Institute, Jeonbuk National University, Iksan, 54596 Republic of Korea; 4 Graduate School of Medical Science & Engineering, Korea Advanced Institute of Science and Technology (KAIST), Daejeon, 34141 Republic of Korea

**Keywords:** Extracellular signalling molecules, Glycobiology, Drug development, Glycosylation

## Abstract

The serum glycoprotein leucine-rich ɑ-2-glycoprotein 1 (LRG1), primarily produced by hepatocytes and neutrophils, is a multifunctional protein that modulates various signaling cascades, mainly TGFβ signaling. Serum LRG1 and neutrophil-derived LRG1 have different molecular weights due to differences in glycosylation, but the impact of the differential glycan composition in LRG1 on its cellular function is largely unknown. We previously reported that LRG1 can promote both angiogenic and neurotrophic processes under hyperglycemic conditions by interacting with LPHN2. Here, we determined the crystal structure of LRG1, identifying the horseshoe-like solenoid structure of LRG1 and its four N-glycosylation sites. In addition, our biochemical and cell-biological analyses found that the deglycosylation of LRG1, particularly the removal of glycans on N325, is critical for the high-affinity binding of LRG1 to LPHN2 and thus promotes LRG1/LPHN2-mediated angiogenic and neurotrophic processes in mouse tissue explants, even under normal glucose conditions. Moreover, the intracavernous administration of deglycosylated LRG1 in a diabetic mouse model ameliorated vascular and neurological abnormalities and restored erectile function. Collectively, these data indicate a novel role of LRG1 glycans as molecular switches that can tune the range of LRG1’s cellular functions, particularly the LRG1/LPHN2 signaling axis.

## Diabetes: Protein structure hints at potential treatment

Resolving the crystal structure of a multifunctional protein suggests possible routes to alleviating symptoms of advanced diabetes. Leucine-rich α-2-glycoprotein 1 (LRG1) plays both positive and negative roles in health and disease. LRG1 glycosylation, where carbohydrate-based polymers known as glycans bind to the protein, alters LRG1’s properties and functions. Aberrant LRG1 glycosylation is recognised in multiple diseases, including diabetes. Jimin Yang at the Institute for Basic Science, Daejeon, South Korea, and co-workers resolved the crystal structure of LRG1, demonstrating its horseshoe shape and identifying four glycosylation sites. Removing glycans from one particular site is vital for the interaction of LRG1 with cellular receptor, LPHN2. This interaction promotes vascular and neurological functioning under high-glucose conditions. When the team used this de-glycosylated LRG1 in a diabetic mouse model, they observed a reduction in several symptoms including erectile dysfunction.

## Introduction

Leucine-rich α-2 glycoprotein (LRG1) is a serum glycoprotein (10–50 μg/mL) primarily produced by hepatocytes^1,2^. Although it was the first member of the large protein family containing leucine-rich repeats (LRRs) to be identified, the physiological role of LRG1 remains poorly understood due to the lack of overt phenotypic abnormalities in *Lrg1*^*−/−*^ mice^1–4^. As the serum concentrations of LRG1 are elevated in patients with various diseases, including cancer, diabetes, cardiovascular disease, and inflammatory disorders^5^, LRG1 has received attention as a prognostic/diagnostic biomarker in these diseases. Reportedly, LRG1 can also be secreted from endothelial cells, epithelial cells, fibroblasts, and other types of myeloid cells in local tissue, such as the lung, kidney, heart, skin, brain, and testis^3^. LRG1 in these local tissue microenvironments is implicated in disease pathogenesis, including tumor metastasis and aberrant neovascularization in chronic obstructive pulmonary disease, diabetic retinopathy, and diabetic nephropathy^4,6–9^. LRG1 exerts its pathogenic functions by modulating multiple transduction cascades, mainly the TGFβ signaling pathway.

Paradoxically, emerging data in a recent study suggest beneficial functions of LRG1 in the acute response to infection and injury^3^. In particular, upregulated LRG1 expression in response to bacterial infection can mediate the differentiation and infiltration of lymphocytes and promote the survival of circulating immune cells by neutralizing cytochrome c cytotoxicity^10–15^. In addition, LRG1 promotes wound healing or tissue repair by stimulating the renewal of damaged epithelial cells, tissue vascularization, and peripheral nerve regeneration^16–19^. Recently, our preclinical studies in mouse models of diabetic erectile dysfunction (ED) showed that LRG1 administration into penile tissue effectively restores erectile function by rescuing vascular and neurological abnormalities through the activation of the LPHN2 axis, which is a TGF-β-independent receptor for LRG1^20^. Thus, LRG1 is a multifunctional protein with context-dependent effects.

LRG1 can also be synthesized in primary human neutrophils during neutrophilic granulocyte differentiation and is then released extracellularly after neutrophil activation to modulate the microenvironment. Interestingly, neutrophil-derived LRG1 undergoes further posttranslational modifications, namely, glycosylations, accounting for its higher molecular weight (~60 kDa) compared to serum-derived LRG1 (~50 kDa)^10^. However, neutrophil-derived and serum LRG1 bind to cytochrome c at similar levels. How the glycosylation or deglycosylation of LRG1 is regulated in vivo and the impact of differential glycosylation patterns in LRG1 on its cellular function are unclear. In this study, we determined the crystal structure of LRG1, identifying the detailed molecular architecture and four N-glycosylation sites of LRG1. Moreover, we found that the deglycosylation of LRG1, particularly the removal of glycans from N325, converts LRG1 to a more potent angioneurin by enhancing the affinity of LRG1 for LPHN2 and thus the subsequent TGF-β-independent signaling. New insights into the structure of LRG1 and the functional significance of glycans can be applied to the development of therapies for diabetic complications associated with endothelial dysfunction and neuropathy.

## Materials and methods

### Recombinant protein expression and purification

Recombinant human LRG1 protein and human LPHN2 variant proteins (Lec, Olf, Lec/Olf domain, or ecto-full domain) were expressed and purified as described previously^20^. The constructs used for this study are listed in Supplementary Table 4. LRG1 N-glycan mutants (N79D, N186D, N269D, and N325D) were constructed using the QuikChange Lightning Site-Directed Mutagenesis kit (#210518, Agilent Technologies) with the LRG1-Fc construct as a template. DG-LRG1 was prepared by incubating LRG1 with GST-fused PNGase F at 4 °C overnight and then passing the solution over GST-Sepharose beads to remove GST-fused PNGase F.

### Crystallization and structure determination

Crystallization and diffraction data collection were performed as described previously^21^. The LRG1 crystal belonged to space group *P*6_3_22 and had the following unit cell dimensions: *a* = 143.0 Å; *b* = 143.0 Å; *c* = 113.7 Å; *α* = 90°; *β* = 90°; and *γ* = 120°. The initial phases were calculated by molecular replacement using PHASER^22^, and the structure of NGL3 (Protein Data Bank code: 3ZYN) was used as a search probe for structure determination. The atomic model was built after iterative rounds of model building using the program COOT^23^ and refinement using the program PHENIX^24^. The final model was validated using MolProbity in PHENIX (*R*_work_ = 18.58%/*R*_free_ = 21.94%). A Ramachandran plot analysis of the LGR1 structure showed that 92.51% and 0.00% of residues were in favored and outlier regions, respectively. The data collection and refinement statistics are shown in Supplementary Table 1. The structure factor and coordinate files have been deposited in the Protein Data Bank under accession code 8H24. All figures for structural models were generated using PyMOL (v2.3.1)^25^.

### Solid-phase binding assay

LPHN2 extracellular domain variants (Lec, Olf, Lec/Olf domain, or ecto-full domain; 100 nM) were added to MaxiSorp 96-well plates (Nunc) and incubated for 1 hour at room temperature. Wells were washed twice with PBS and incubated with 1% bovine serum albumin (BSA) for 2 hours. After blocking, varying amounts (0.001, 0.005, 0.01, 0.05, 0.1, 0.5, 1, 5, or 10 μM) of DG-LRG1 or LRG1 N-glycan mutants (N79D, N186D, N269D, and N325D) were added to 96-well plates coated with the indicated LPHN2 extracellular domain variants. We detected DG-LRG1 or LRG1 N-glycan mutants (N79D, N186D, N269D, and N325D) bound to coated LPHN2 proteins by ELISA using an anti-LRG1 antibody (Cat# sc517443, Santa Cruz) and peroxidase-conjugated anti-mouse secondary antibody (Cat# 62-6520, Thermo Fisher Scientific).

### Cell culture and lentivirus treatments

Human umbilical vein endothelial cells (HUVECs) were purchased from Lonza (Cat# CC-2519). HUVECs were cultured in EBM-2 (Cat# CC-3156, Lonza) supplemented with EGM-2 (Cat# CC-3162, Lonza), 2 mM L-glutamine (Cat# 25030081, Gibco^TM^), 100 U/ml penicillin, and 100 μg/ml streptomycin (Cat# 15140122, Gibco^TM^) on gelatin (Cat# G1890, Sigma‒Aldrich; 0.1% in PBS)-precoated plates in 5% CO_2_ in a humidified incubator. The dorsal root ganglia (DRG) were isolated from C57BL/6 mice and digested as described previously^26^. DRGs were plated onto poly-D-lysine (Cat# A3890401, Thermo Fisher Scientific)-coated dishes in Minimum Essential Medium (Cat# 11095080, Thermo Fisher Scientific) containing N-2 Supplement (Cat# 17502048, Thermo Fisher Scientific), 2 mM L-glutamine (Cat# 25030081, Thermo Fisher Scientific), 100 U/ml penicillin, and 100 μg/ml streptomycin (Cat# 15140122, Gibco^TM^). Cells were cultured in a humidified 37 °C incubator with 5% CO_2_. For LPHN2 knockdown in HUVECs and DRG neurons, shLPHN2 lentivirus particles were added to the culture medium at 5 × 10^4^ TU/ml as described previously^20^. The scrambled shRNA was used as a control.

### Apparent binding affinity of LRG1 to HUVECs and HEK293T cells

The apparent binding affinity of LRG1 for parental and LPHN2-knockdown HUVECs was measured by treating 1.0 × 10^6^ cells/200 µl with 1 μM Alexa 647-conjugated LRG1 or Alexa 647-conjugated DG-LRG1. After washing with PBS, cell surface-bound LRG1 or DG-LRG1 was detected by monitoring fluorescence signals by fluorescence-activated cell sorting (FACS) and analyzed using FlowJo 10 software (FlowJo, LLC).

### Tube formation and cell migration assay

The tube formation assay with HUVECs was performed as described previously^27^. After treating HUVECs with 1 μg/ml LRG1, DG-LRG1, or LRG1 N-glycan mutants (N79D, N186D, N269D, and N325D), tube formation was monitored for 12–16 hours under a phase-contrast microscope and quantified by counting the number of master junctions from four separate experiments in a blinded manner using ImageJ 1.34. Migration assays were performed using a modified Boyden chamber with polycarbonate filters (8 μm pores, Corning) coated with 0.1% gelatin. A total of 1 × 10^5^ HUVECs in 200 μl of serum-free medium were seeded in the upper chamber of 12-well plates, and 600 μl of culture medium was added to the lower chamber^28^. HUVECs were treated with 1 μg/ml LRG1, DG-LRG1, or LRG1 N-glycan mutants (N79D, N186D, N269D, and N325D). After culturing for 18 hours at 37 °C, nonmigrating cells on the upper surface of the insert were removed with a cotton swab. Migrated cells on the bottom surface of the filter were fixed with 4% formaldehyde, stained with crystal violet (Cat# V5265, Sigma), and counted at ×400 magnification under a microscope.

### DRG explant culture and sprouting assay

Mouse DRGs were dissected and maintained as described previously^20^. After DRG culture was treated separately with LRG1 (1 µg/ml) or DG-LRG1 (1 µg/ml) or one of the LRG1 mutants (N79D, N186D, N269D, N325D) for 7 days, neurite outgrowth segments were fixed in 4% paraformaldehyde for at least 30 minutes and immunostained with anti-βIII-tubulin (Cat# ab107216, Abcam; 1:100).

### Western blot analysis

Cells were lysed in RIPA lysis buffer containing 1X protease inhibitor cocktail (Cat# P3100, GenDEPOT) and phosphatase inhibitors (Cat# P3200, GenDEPOT). Proteins were resolved via SDS‒PAGE and transferred onto PVDF membranes (Cat# 1620177, Bio-Rad). The membranes were incubated in the presence of the primary antibody overnight at 4 °C and then with appropriate HRP-conjugated secondary antibodies (Thermo Fisher Scientific). The antibodies chosen for this western blot analysis are listed in Supplementary Table 4: anti-LPHN2 (Cat# MBS244156, MyBioSource), anti-LRG1 (Cat# HPA001888, Sigma‒Aldrich), anti-phospho-Akt (Cat#9271, Cell Signaling Technology), anti-Akt (Cat# 9272, Cell Signaling Technology), anti-phospho-PI3 kinase p85 (Cat#4282, Cell Signaling Technology), anti-PI3 kinase p85 (Cat# 4292, Cell Signaling Technology), anti-NF-kB (Cat #8242, Cell Signaling Technology), phospho-NF-kB (Cat# MA5-15181, Thermo Fisher Scientific), and an internal control (β-actin; Cat# sc-69879, Santa Cruz Biotechnology).

### Animals and measurement of erectile function

Eight-week-old male C57BL/6 (Orient Bio, Korea) mice were used in this study. The mice were age-matched in all experiments. Experiments were conducted with approval from the Inha University Animal Care and Use Committee (Assurance Number: INHA 180523-570). Diabetes was induced by intraperitoneal injection of streptozotocin (STZ; 50 mg/kg body weight) for 5 consecutive days as described previously^29^. Eight weeks after diabetes induction, the mice were anesthetized with intramuscular injections of ketamine (100 mg/kg) and xylazine (5 mg/kg). Erectile function was measured as described previously^29^. Briefly, the maximal intracavernous pressure (ICP) was recorded during stimulation (5 V at a frequency of 12 Hz, pulse width of 1 ms, and duration of 1 minute) using bipolar platinum wire electrodes placed around the cavernous nerve. The total ICP was determined as the area under the curve from the beginning of cavernous nerve stimulation to a point 20 seconds after stimulus termination. Systemic blood pressure was measured using a noninvasive tail-cuff system (Visitech Systems) prior to measuring the ICP. The ratios of the maximal ICP and total ICP to the mean systolic blood pressure (MSBP) were calculated to account for variations in the systemic blood pressure.

### Histological examination of immunofluorescence

Mouse penile tissues were fixed in 4% paraformaldehyde for 10 minutes at room temperature and then blocked in 1% BSA (Cat# 9048-46-8, Sigma‒Aldrich) for 2 hours at room temperature. Immunohistochemistry was performed as described previously^20^ using primary antibodies [anti-PECAM1 antibody (Cat# MAB1398Z, Millipore), anti-NG2 antibody (Cat# ab5320, Millipore), anti-βIII-tubulin antibody (Cat# ab107216, Abcam)] and secondary antibodies [goat anti-Armenian hamster fluorescein (FITC) (Cat# 127-095-160, Jackson ImmunoResearch Laboratories), donkey anti-rabbit DyLight® 550 (Cat# ab98489, Abcam), and donkey anti-chicken rhodamine (TRITC) (Cat# 703-025-155, Jackson ImmunoResearch Laboratories)]. Immunofluorescence staining intensity was quantified using ImageJ 1.34.

### Statistical analysis

All data are presented as the mean ± SEM. For cell culture data, three independent experiments were performed in triplicate. For normally distributed data, a two-tailed Student’s *t* test was used to compare the two groups. For comparisons among groups, one-way ANOVA and post hoc Bonferroni testing were performed using Prism 9 software (Graph Pad Software). A *p* value < 0.05 was considered significant.

## Results

### Crystal structure of LRG1 and identification of the N-glycosylation site

**Fig. 1:**
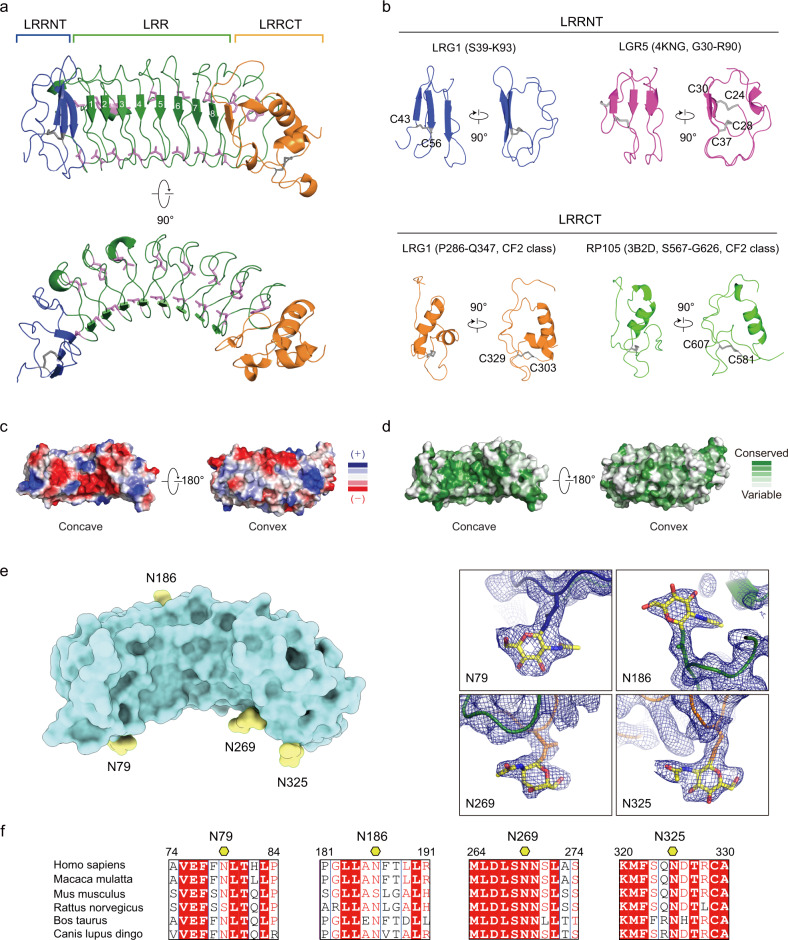
Crystal structure of LRG1 and its N-glycosylation site. **a** Overall structure of human LRG1 (top and side views). Blue, LRRNT; green, eight LRR modules; orange, LRRCT; gray, disulfide bridge; magenta, phenylalanine spine and asparagine ladder. **b** Structural comparison of the LRRNT and LRRCT domains of human LRG1 to those of other LRR proteins. Disulfide bridges are presented as gray lines. **c** Electrostatic potential of LRG1 calculated according to the Poisson-Boltzmann equation in PyMOL. Blue and red on the surface of the LRG1 structure represent positively and negatively charged residues, respectively. The orientation of the concave view is identical to that in **a** (top). **d** Sequence conservation was calculated by the Consurf server^54^ and is presented on the surface of LRG1. The structures are shown as surface representations, with color maps reflecting conservation (green, highly conserved; white, less conserved). The orientation of the surface view of LRG1 is identical to that in **c**. **e** Location of glycans (yellow) attached to human LRG1 (cyan surface). The right panel depicts the electron density map (2Fo-Fc map) contoured at 2σ for N-acetylglucosamine, which is attached to asparagine residues (N79 on LRRNT, N186 on the convex surface of LRR repeat, N269 on the concave surface of the LRR repeat, and N325 on LRRCT). **f** Sequence alignment of glycosylation sites on LRG1. Red box with white characters, strict identity; red characters, similarity within a group; black characters, similarity across groups. Glycosylated asparagine residues are indicated by yellow hexagons.

LRR domains typically form horseshoe shapes and are considered a suitable structural motif for protein‒protein interactions^30,31^. To delineate the structure of LRG1 and its glycans, we expressed full-length human LRG1 (NCBI accession number: NP_443204, residues V36-Q347) using Freestyle 293-F cells^21^ and determined the crystal structure of LRG1 at 2.5 Å resolution (Fig. 1a). Crystallographic details are summarized in Supplementary Table 1. The structure of LRG1 was solved by molecular replacement using NetrinG ligand 3 (PDB code: 3ZYN) as an initial search probe^32^, which has a sequence similar to that of LRG1 (sequence identity, 27.24%). The refined structure of LRG1 adopts a horseshoe-like solenoid structure that includes eight central LRR repeats (LRR1-LRR8) flanked by LRRNT and LRRCT domains (Fig. 1a). Similar to other typical LRR family members, each LRR repeat of LRG1 possesses a conserved LxxLxLxxNxL motif (where ‘x’ is any amino acid) composed of 24 amino acids. The concave face consists of parallel β-strands, whereas the convex surface comprises short 3_10_ helices (R113-V115, V157-L160) or connecting loops. A sequence alignment of mammalian LRG1s indicates that the critical residues (consensus leucine residues and an asparagine ladder in the LxxLxLxxNxL motif, a phenylalanine spine, and four cysteines in LRRNT and LRRCT) are all highly conserved (Supplementary Fig. 1).

LRRNT and LRRCT, which are usually stabilized by two respective disulfide bonds, shield the hydrophobic cores of the LRR motifs. However, the structures of LRRNT and LRRCT in LRG1 are slightly different from those in a typical LRR family protein. The LRRNT of LRG1 contains three β-strands, and its N-terminal β-hairpin is stabilized by only a single disulfide bridge that connects C43 and C56, whereas the LRRNT of other LRR families usually contains two disulfide bridges (Fig. 1b, top). The LRRCT of LRG1 contains one disulfide bond that connects C303 and C329 (Fig. 1b, bottom); thus, LRG1 belongs to the CF2 class^30^.

Cytochrome c was found to bind LRG1^33,34^. The three-dimensional structures of the complexes between LRR proteins and proteins that bind them, such as variable lymphocyte receptors (VLRs), synaptic adhesion protein (Slitrk), and acid-labile subunit (IGFALS), have shown that the binding proteins are surrounded at least in part by the concave surface of the LRR domain, indicating that the elongated and curved LRR structure provides an outstanding framework for diverse protein‒protein interactions^35–38^. Therefore, the radius of curvature and unique properties of the concave surface of LRR proteins are critical determinants for specific LRR protein-binder complexes. Accordingly, the conserved charged residues in the concave surface of LRG1 are likely responsible for the binding of cytochrome c (Fig. 1c, d and Supplementary Table 2). The structure prediction of the LRG1-cytochrome c complex using AlphaFold-multimer^39^ and the molecular docking of cytochrome c to LRG1 using Cluspro^40^ indicate that the conserved negatively charged residues in the concave surface of LRG1 (E76, E96, S100, D122, D194, E220, D266, S268, and D295) interact with the positively charged residues of cytochrome c (K9, K14, and K73) (Supplementary Fig. 2a–c).

LRG1 contains ~23% carbohydrates by weight and is predicted to contain five glycosylation sites (4 N- and 1 O-linked glycosylation sites, N79, N186, N269, N325 and T37, respectively)^2,41^. The electron density map of the LRG1 crystal structure and OMIT maps for glycans calculated by PHENIX^42^ clearly showed that N-acetylglucosamines are attached to four asparagine residues: N79 on LRRNT, N186 on the convex surface of LRR repeats, N269 on the concave surface of LRR repeats, and N325 on LRRCT (Fig. 1e and Supplementary Fig. 2d). The glycans attached to N79, N269, and N325 were located on the same concave surface; among them, both N269 and N325 are highly conserved (Fig. 1f). However, O-linked glycosylation on T37 was not observed, because we could not build the three flexible residues (V36-L38) of LRG1.

### Deglycosylation of LRG1 enhances its binding affinity toward LPHN2 and its angiogenic and neurotrophic functions

**Fig. 2:**
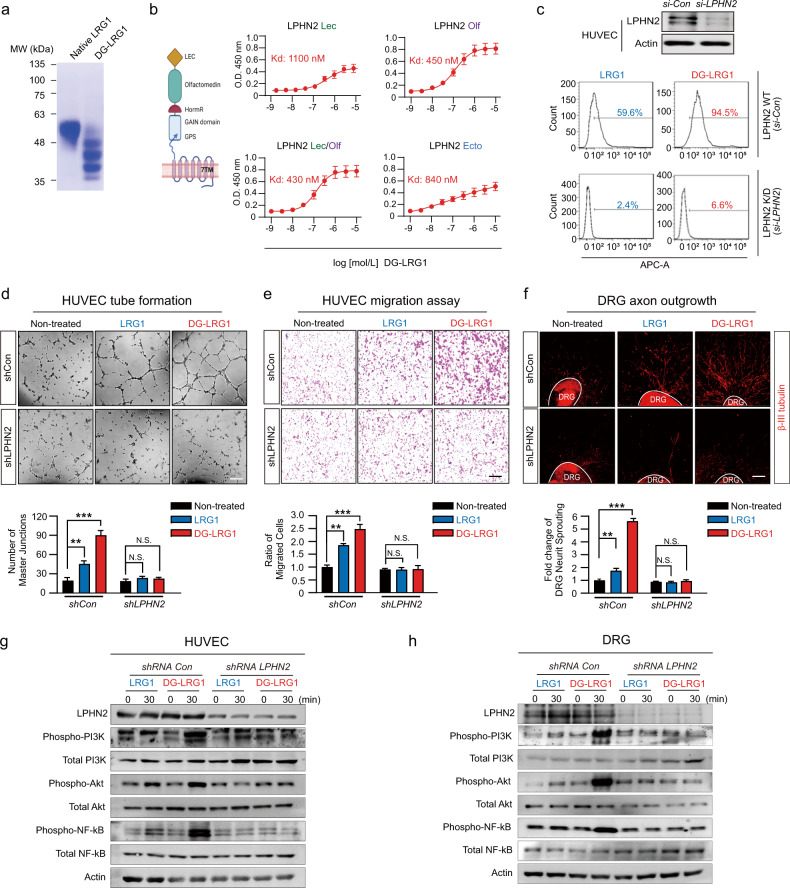
Deglycosylated LRG1 potentiates angiogenesis and neurite outgrowth through increased binding to LPHN2. **a** SDS‒PAGE of recombinant LRG1 and DG-LRG1 (incubation of LRG1 with 40 µg/ml PNGase F at 4 °C overnight). **b** Left, schematic of the domain architecture of LPHN2. Lec, lectin; Olf, olfactomedin-like; HomoR, hormone receptor motif; GAIN/GPS, GPCR autoproteolysis-inducing/GPCR proteolysis site. Right, the binding of DG-LRG1 to the LPHN2 ectodomain (Lec, Olf, Lec+Olf, or Ecto-full domain) was determined by a solid-phase binding assay. **c** Binding of Alexa 647-conjugated LRG1 or DG-LRG1 (1 μM) to parental and LPHN2-knockdown HUVECs was analyzed by FACS. **d**, **e** Tube formation assay (**e**) and Transwell cell migration assay (**f**) after treatment of HUVECs with LRG1 (1 μg/ml) or DG-LRG1 (1 μg/ml) in the presence of shControl or shLPHN2 lentivirus (5 × 10^4^ TU/ml culture medium). Top, representative images of tube formation and Transwell cell migration. Scale bars, 200 µm. Bottom, quantification of the number of master junctions and migrated cells using ImageJ. **f** βIII-tubulin immunostaining in mouse DRG explants after treatment with LRG1 (1 μg/ml) or DG-LRG1 (1 μg/ml) in the presence of shCon or shLPHN2 lentivirus (5 × 10^4^ TU/ml culture medium). Top, representative images of DRG explants. Scale bars, 200 µm. Bottom, quantification of βIII-tubulin-immunopositive neurite length from DRG explants (200 cells/field) using ImageJ. **d**, **f** Data in bar graphs are plotted as the mean ± SEM (*n* = 4). Significance is indicated using Student’s *t* test (**p < 0.01; ***p < 0.001). The relative ratio of the nontreated group was defined as 1. Western blot with HUVECs (**g**) and mouse primary DRG explants (**h**) after treatment with LRG1 (1 μg/ml) or DG-LRG1 (1 μg/ml) in the presence of shCon vs. shLPHN2 lentivirus (*n* = 3).

Recently, we reported that the adhesion GPCR latrophilin-2 (LPHN2) is a TGF-β-independent receptor of LRG1, and the LRG1/LPHN2 axis exerts both angiogenic and neurotrophic effects under hyperglycemic conditions^20^. Given that secreted LRG1 can be differentially glycosylated and that alterations in sugar chains may influence the function of LRG1 in cancer^43,44^, we investigated the functional significance of LRG1’s N-glycans on the LRG1/LPHN2 axis. To this end, we first examined whether the deglycosylation of LRG1 (DG-LRG1) affects its binding affinity to LPHN2 using in vitro solid-phase binding assays. On SDS‒PAGE, an approximately 50 kDa band for native LRG1 was reduced to a 35–45 kDa heterogeneous mixture by PNGase F treatment (Fig. 2a). Similar to native LRG1^20^, DG-LRG1 was able to bind to the olfactomedin-like (Olf) domain alone and to lectin (Lec) + Olf domain, confirming that the Olf domain is the minimal LPHN2 domain for binding native LRG1 or DG-LRG1 (Fig. 2b). Surprisingly, the binding affinities of LRG1 WT for the LPHN2 Lec/Olf domain (K_D_ = 920 nM) and ecto-full (no binding) were markedly increased by the deglycosylation of LRG1 (Lec/Olf domain K_D_ = 450 nM; ecto-full domain K_D_ = 840 nM). These results were also confirmed by assessing the apparent cell surface binding of LRG1 and DG-LRG1 in HUVECs (59.6% for LRG1 vs. 94.5% for DG-LRG1) (Fig. 2c). Moreover, the cell surface binding of LRG1 or DG-LRG1 was dramatically diminished in LPHN2-knockdown HUVECs (2.4% for LRG1 vs. 6.6% for DG-LRG1) (Fig. 2c). However, DG-LRG1 could not bind to the Lec/Olf domain of LPHN1 or LPHN3, similar to LRG1, despite 87% sequence similarity to the Lec/Olf domain of LPHN2 (Supplementary Figs. 3a, 4a).

We then examined the angiogenic and neurotrophic effects of DG-LRG1 on HUVECs and DRG explants. Unlike native LRG1, which functions under hyperglycemic conditions, DG-LRG1 significantly promoted the tube formation and cell migration of HUVECs (Fig. 2d, e) and axonal sprouting from DRG explants (Fig. 2f), even under normal glucose conditions. However, LPHN2 knockdown with shLPHN2 lentivirus in HUVECs and DRG explants markedly reduced these effects (Fig. 2d–f). As PI3K, AKT, and NF-κB p65 signaling constitute the key signaling pathway for LRG1/LPHN2-mediated angiogenesis and neurite outgrowth under hyperglycemic conditions^20^, we next compared these signaling pathways upon treatment of HUVECs and DRG with native LRG1 or DG-LRG1. Consistent with the above phenotypic outcomes, DG-LRG1 robustly increased the phosphorylation levels of PI3K, AKT, and NF-κB under normal glucose conditions, and the phosphorylation of these signaling components upon treatment with DG-LRG1 was markedly reduced by shRNA-mediated LPHN2 knockdown (Fig. 2g, h). These results indicate that the deglycosylation of LRG1 enhances its binding affinity to the LPHN2 receptor, thus promoting LPHN2-mediated angiogenesis and neurite outgrowth even under normal glucose conditions through the same cellular signaling pathway as LRG1 does under hyperglycemic conditions.

### N325 is a crucial glycosylation site for attenuating LRG1 function under normal glucose conditions

**Fig. 3:**
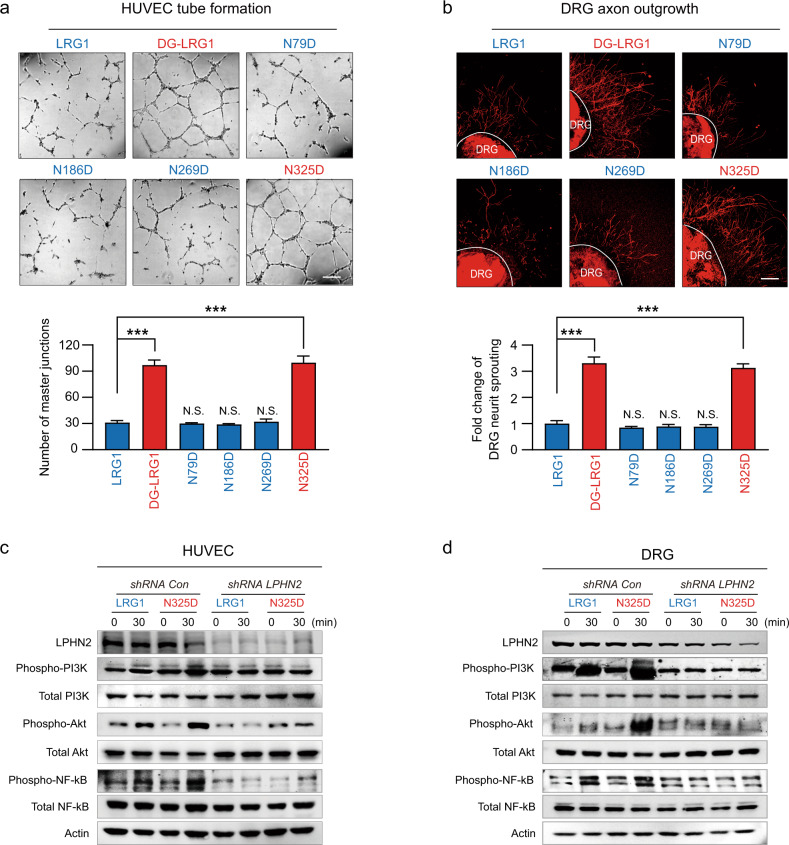
LRG1 N325 is the key glycosylation site for attenuating LRG1/LPHN2-mediated angiogenesis and neurite outgrowth. **a** Tube formation assay using HUVECs after treatment with 1 μg/ml LRG1, DG-LRG1, or LRG1 glycan mutants (N79D, N186D, N269D, N325D). Representative images of tube formation and Transwell cell migration. Scale bars, 200 µm (top). Bottom, quantification of the number of master junctions and migrated cells using ImageJ. **b** βIII-tubulin immunostaining in mouse DRG explants after treatment with 1 μg/ml LRG1, DG-LRG1, or LRG1 glycan mutants (N79D, N186D, N269D, N325D). Representative images of DRG explants. Scale bars, 200 µm (top). Bottom, quantification of βIII-tubulin-immunopositive neurite length from DRG explants (200 cells/field) using ImageJ. In the bar graphs, data are plotted as the mean ± SEM (*n* = 4). Significance is indicated using Student’s *t* test (***p < 0.001). The relative ratio of the nontreated group was defined as 1. N.S., not significant. Western blot with HUVECs (**c**) and mouse primary DRG explants **d** after treatment with LRG1 (1 μg/ml) or LRG1 N325D mutant (1 μg/ml) in the presence of shCon vs. shLPHN2 lentivirus (*n* = 3).

To determine the glycan(s) responsible for attenuating the angiogenic and neurotrophic activity of LRG1, we mutated individual glycosylation sites to aspartic acid, which mimics the deglycosylated form of asparagine (Supplementary Fig. 3b). After we confirmed that the mutation of each glycosylation site of LRG1 does not affect the level of protein expression, we monitored HUVEC tube formation and neurite outgrowth with DRG explants upon treatment with these mutant variants. Intriguingly, only the LRG1 N325D mutant could induce tube formation and neurite outgrowth to a similar extent to DG-LRG1, even under normal glucose conditions (Fig. 3a, b), suggesting that the effects of DG-LRG1 are mainly attributable to detachment of glycan at the highly conserved N325 residue. We also confirmed that DG-LRG1 and N325D have higher binding affinity to the Lec/Olf domain of LPHN2 than LRG1 WT, N79D, N186D and N269D using a solid-phase binding assay (Fig. 2b and Supplementary Fig. 3c).

Next, we examined whether the LRG1 N325D mutant can activate the LRG1/LPHN2-mediated intracellular signaling pathways responsible for promoting angiogenic and neurotrophic effects. Similar to DG-LRG1, treatment with the LRG1 N325D mutant significantly increased the phosphorylation of PI3K-AKT-NF-κB in HUVECs and DRG under normal glucose conditions, and LPHN2 knockdown diminished the phosphorylation level of those proteins (Fig. 3c, d). Taken together, our data indicate that glycans on the conserved N325 residue are pivotal for attenuating LRG1/LPHN2-mediated angiogenesis and neurite outgrowth under normal glucose conditions. In addition, the data suggest that the removal of glycan from N325 by an unknown factor under pathophysiological conditions may convert fully glycosylated LRG1 to a more angiogenic and neurotrophic form.

### DG-LRG1 and the LRG1 N325D mutant restore erectile function through dual angiogenic and neurotrophic effects in diabetic mice

**Fig. 4:**
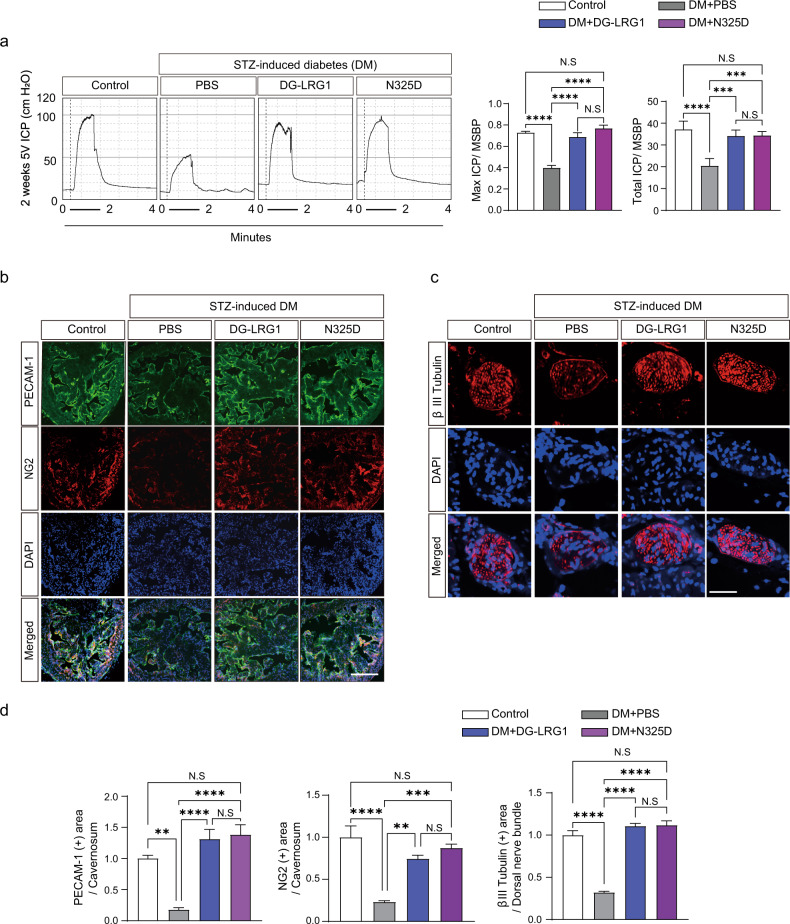
Deglycosylation of LRG1 and the LRG1 N325D mutant restores erectile dysfunction under diabetic conditions. **a** Representative intracavernous pressure (ICP) responses in STZ-induced diabetic mice and age-matched nondiabetic controls 2 weeks after repeated intracavernous injection of PBS (negative control), DG-LRG1 (5 µg/20 µl), or the LRG1 N325D mutant (5 µg/20 µl) on Days 0 and 3. The cavernous nerve was stimulated at 5 V. The stimulus interval is indicated by a solid bar. The ratios of the mean maximal ICP and total ICP (area under the curve) to mean systolic blood pressure (MSBP) were calculated for each group (*n* = 5). **b** Representative immunofluorescence (IF) of platelet/endothelial cell adhesion molecule-1 (PECAM-1, green) and neuron-glial antigen 2 (NG2, red) in cavernous tissue from control and STZ-induced diabetic mice 2 weeks after repeated intracavernous injection of PBS (negative control), DG-LRG1 (5 µg/20 µl), or LRG1 N325D mutant (5 µg/20 µl) on Days 0 and 3. Scale bar, 100 µm. **c** Representative IF of βIII-tubulin (red) in dorsal nerve bundle (DNB) tissue from control and STZ-induced diabetic mice 2 weeks after repeated intracavernous injections as indicated in **b**. Scale bars, 25 µm. Nuclei were stained with DAPI (blue). **d** The PECAM-1 (green), NG (red), or βIII-tubulin (red)-immunopositive areas in the corpus cavernosum and dorsal nerve bundle of control and diabetic mice were quantified by ImageJ (*N* = 5 and 6, respectively). Data are plotted as the mean ± SEM. Significance is indicated using one-way ANOVA with Bonferroni’s multiple comparisons test (*p < 0.05, **p < 0.01, ***p < 0.001, ****p < 0.0001).

The pathophysiological mechanism underlying diabetic ED reflects endothelial dysfunction and autonomic neuropathy^45,46^. We previously reported that the intracavernous delivery of recombinant LRG1 restores erectile function in STZ-induced diabetic mice by enhancing endothelial and cavernous nerve regeneration^20^. To test the potent efficacy of DG-LRG1 and LRG1 N325D, we measured ICP upon electrical stimulation of the cavernous nerve in diabetic mice after intracavernous injection of DG-LRG1 or LRG1 N325D. The ratios of maximal ICP and total ICP to MSMP were significantly lower in saline-treated diabetic mice than in age-matched controls. MSBP did not differ among the experimental groups (Supplementary Table 3). Notably, repeated intracavernous injection of DG-LRG1 or LRG1 N325D restored the erection parameters in diabetic mice to ≤92% of the control values (Fig. 4a). Next, we performed immunofluorescence staining of platelet/endothelial cell adhesion molecule (PECAM)-1, the pericyte marker neuronal-glial antigen 2 (NG2) in the corpus cavernosum, and βIII-tubulin in dorsal nerve bundles (DNBs). Consistent with a previous study using native LRG1, the intracavernous administration of DG-LRG1 or LRG1 N325D to diabetic mice rescued the decreased numbers of cavernous endothelial cells and pericytes (Fig. 4b, d) and restored neurofilaments at DNBs (Fig. 4c, d). These results imply that the local administration of DG-LRG1 or the LRG1 N325D mutant could be a promising therapeutic strategy for treating diabetic ED.

## Discussion

LRG1 is a multifunctional protein that can modulate various signaling cascades, including TGFβ canonical/noncanonical signaling and LPHN2 signaling. Interestingly, serum LRG1 and neutrophil-derived LRG1 have different glycosylation patterns, and aberrant glycosylation patterns on LRG1 have been observed in cancer patients^3^. Here, our in-depth structural and functional analysis of LRG1 demonstrates that N-linked glycans at the N325 residue contribute to the attenuation of intermolecular interactions with its cognate receptor, LPHN2. Whereas native LRG1 functions as an angioneurin under hyperglycemic conditions, the deglycosylation of LRG1 is essential for high-affinity binding between LRG1 and LPHN2 and the subsequent activation of LPHN2-mediated signaling even under normal glucose conditions.

LRG1, an LRR protein family member, comprises LRRNT, eight LRR repeats, and LRRCT. Our crystal structure showed that LRG1 has the characteristic curved solenoid structure of LRR proteins with the hydrophobic core of the LRR solenoid and a concave surface consisting of parallel β-strands. AlphaFold-multimer allowed us to predict the complex structure of LRG1 and cytochrome c, whose orientation is similar to the docking results from ClusPro using our LRG1 structure and cytochrome c [PDB ID: 3ZCF]^39,40,47^. These findings suggest that our prediction of the LRG1/cytochrome c complex is relatively accurate and that the concave surface of LRG1, particularly the conserved negatively charged residues on the concave surface, is the most likely binding site for cytochrome c. Moreover, the potential binding site for cytochrome c does not overlap with the position of the N-linked glycans on LRG1, explaining why the differentially glycosylated LRG1 from neutrophils exhibits similar binding properties to serum LRG1^10^. However, the crystal structure of LRG1 in complex with cytochrome c or TGFβ will be required for further analysis.

Protein glycosylation is a posttranslational modification that plays important roles in protein folding, solubility, stability, activity, subcellular localization, and protein‒protein interactions^48^. O-acetyl–modified terminal sialic acid is known to block the binding of some influenza viruses, whereas removal of the sialic acid itself unmasks the binding site^49^. The circulating half-life of antibodies and their effector functions can be profoundly influenced by relatively subtle changes in the glycosylation state of the IgG Fc regions. Glycans and glycan-modifying enzymes can also modulate receptor‒ligand interactions and subsequent signal transduction. For example, sulfatases (Sulfs) in the extracellular matrix catalyze the 6-O-desulfation of heparan sulfate (HS) on heparan sulfate proteoglycan (HSPG), thereby modulating the interaction of HS with various bioactive ligands, including Wnt, glial cell-derived growth factor (GDNF), fibroblast growth factor (FGF), hepatocyte growth factor (HGF), VEGF, and TGFβ^50^. Therefore, Sulfs can activate Wnt and GDNF signaling but decrease the activity of FGF, HGF, VEGF, and TGFβ. Our data demonstrate that LRG1 glycans can act as molecular switches or rheostats that “tune” the range of cellular functions of LRG1, particularly the LRG1/LPHN2 signaling axis. However, the cellular and molecular mechanisms responsible for LRG1 deglycosylation under physiological conditions remain unknown. Notably, various glycosylation abnormalities have been found in both type 1 and type 2 diabetes patients, suggesting that the levels and/or activities of glycosylation and deglycosylation enzymes are clinical phenotypes associated with diabetes^51^. Moreover, we recently reported that LRG1 promotes both angiogenic and neurotrophic processes in mouse tissue explants under hyperglycemic conditions^20^. Therefore, we speculate that the altered expression, secretion, and/or activity of an unknown factor in hyperglycemia and diabetes mellitus may induce LRG1 deglycosylation, allowing the activation of LRG1/LPHN2-mediated signaling. Further studies are needed to understand the pathophysiological mechanisms underlying LRG1 deglycosylation.

How glycans attached to LRG1 can attenuate the interaction between LRG1 and LPHN2 and why deglycosylated LRG1 can bind only to LPHN2, not to LPHN1 and LPHN3, remain to be determined. FLRT, one of the LPHN ligands involved in synaptic adhesion, is localized to synaptic membranes and contains an LRR domain that mediates binding between the LPHN Olf domain and Unc5^52^. The previously reported crystal structure of the ternary complex [LPHN3 (Lec-Olf domain), FLRT2 (LRR protein), and Unc5] showed that two LPHN3 molecules (LPHN3A and LPHN3B) bind to one FLRT2, such that LPHN3A and LPHN3B lie close to LRRNT and C-terminal LRRCT domains, respectively, at the concave surface of FLRT2^52^ (Supplementary Fig. 4b). Interestingly, the structure of FLRT2 LRR8-LRRCT is very similar to that of LRG1 LRR6-LRRCT (root mean squared deviation=1.243 Å for the aligned Cα atoms; Supplementary Fig. 4c). These observations partially explain why the detachment of glycans from the conserved N325 residue of LRG1 is critical for the high-affinity binding of LRG1 to LPHN2. Specifically, the glycans on N325 of LRG1 LRRCT likely hinder LPHN2 binding, whereas the detachment of these glycans in a particular condition or a differential glycosylation mechanism relieves this steric hindrance, allowing the tight binding of LRG1 LRRCT to the LPHN2 Olf domain, similar to that in the FLRT2/LPHN3B complex. Furthermore, the DDD loop in LPHN3B critical for FLRT2 and LPHN3B interactions is not conserved across LPHN family members (Supplementary Fig. 4a, b), suggesting that this loop has diverged to mediate specific interactions of LPHN family members with distinct binding partners. A detailed understanding of the molecular mechanism underlying LRG1 binding to LPHN2 and LRG1-mediated LPHN2 activation will require additional structural studies on the LRG1/LPHN2 complex.

The long-term complications of diabetes mellitus include macroangiopathy, microangiopathy, neuropathy, and ED^53^. The unmet needs of current limited therapies for diabetic vascular and neuronal complications have prompted investigations in pursuit of developing new treatment options. Our data show that the administration of DG-LRG1 or the N325 mutant effectively activates cellular signaling via the PI3K-AKT-NF-κB pathway in endothelial cells and neurons, rescuing vessel and peripheral nerve abnormalities, as well as diabetic ED (Fig. 4). On the basis of our current study and previous findings, we propose a model suggesting that serum LRG1 can exert its cellular functions only at specific sites where LRG1 is deglycosylated and/or TGFβ is expressed. Our findings provide new insights into the functional significance of glycans on LRG1 as a molecular switch that tunes the range of LRG1 cellular functions, particularly the LRG1/LPHN2 axis. These can be applied to the development of therapies for diabetic complications associated with endothelial dysfunction and neuropathy.

## Supplementary information

Supplementary Information

Validation Reports for PDB deposit

## Data Availability

The coordinate file and structure factor file for the crystal structure of LRG1 have been deposited in the Protein Data Bank (PDB) under accession code 8H24. All study data are included in the article and/or Supplemental Information. All other data and materials used for the analysis are available from the corresponding author upon reasonable request.
