# Supplementary material for: Crystal structure of LRG1 and the functional significance of LRG1 glycan for LPHN2 activation
Source: Exp Mol Med. 2023 May 1;55(5):1013–22. doi: 10.1038/s12276-023-00992-4 (PMC10238393; doi:10.1038/s12276-023-00992-4)
Supplement: Supplementary file 1 — Supplementary Information [file 12276_2023_992_MOESM1_ESM.pdf]

Supplementary Information

Crystal structure of LRG1 and the functional significance of LRG1 glycan for LPHN2 activation

Jimin Yang, Guo Nan Yin, Do-Kyun Kim, Ah-reum Han, Dong Sun Lee, Kwang Wook Min, Yaoyao Fu, Jeongwon Yun, Jun-Kyu Suh, Ji-Kan Ryu, and Ho Min Kim

This PDF file includes:

Supplementary Figures 1-4

Supplementary Tables 1-4

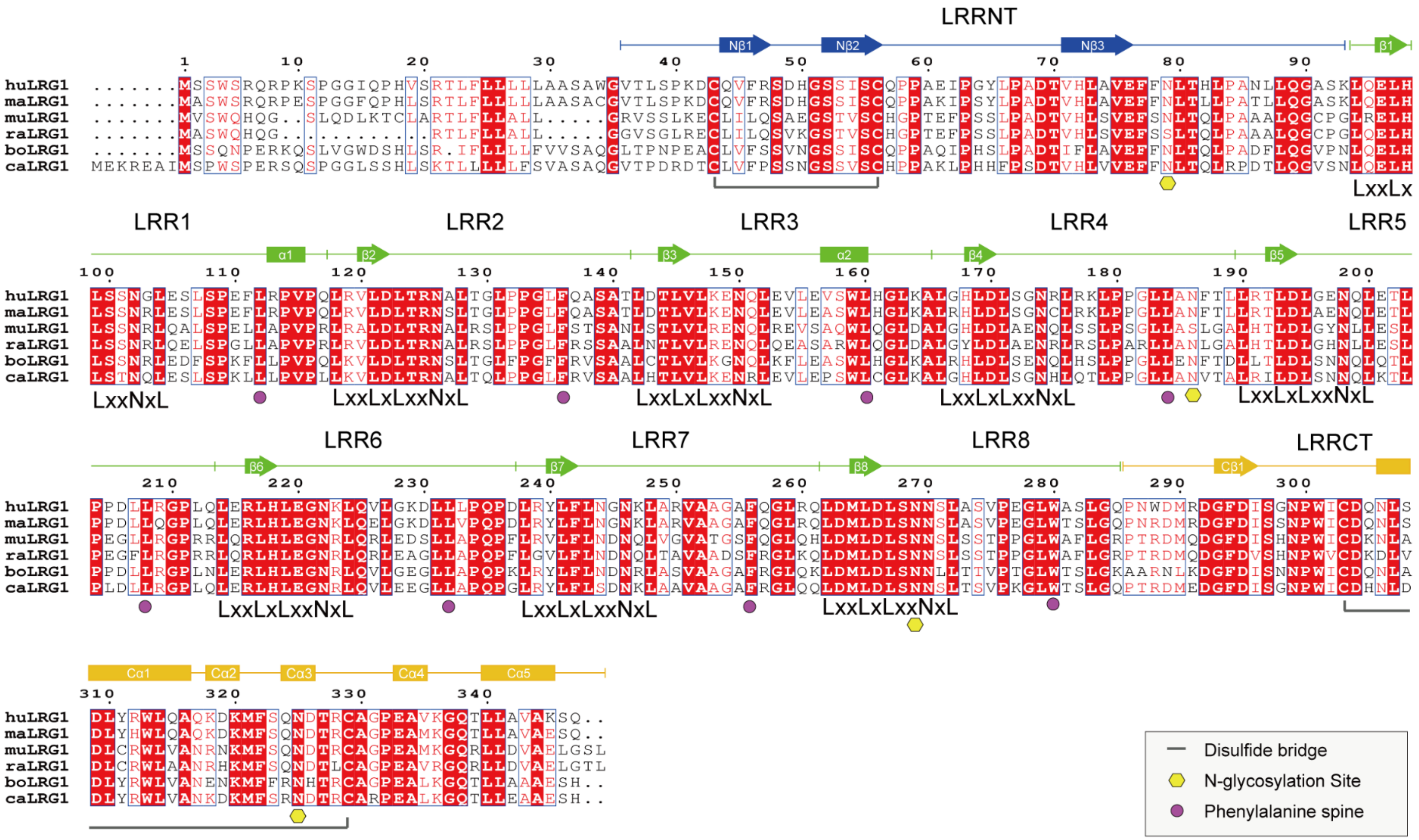

Supplementary Fig. 1 | Sequence alignment of human LRG1

Sequence alignment of LRG1 from *Homo sapiens* (huLRG1, NP_443204.1), *Macaca mulatta* (maLRG1, EHH29497.1), *Mus musculus* (muLRG1, NP_084072.1), *Rattus norvegicus* (raLRG1, NP_001009717.1), *Bos taurus* (boLRG1, DAA27797.1), and *Canis lupus dingo* (caLRG1, XP_025312813.1). Consensus sequences (LxxLxLxxNxL) and key conserved phenylalanine, disulfide bridges, and glycosylated Asn residues are indicated below the sequence alignment. Secondary structure elements are noted above the alignment for β-strands (arrows) and α-helices (cylinders). The sequence alignment was created using T-Coffee (http://tcoffee.crg.cat) and ESPript servers (http://espript.ibcp.fr).

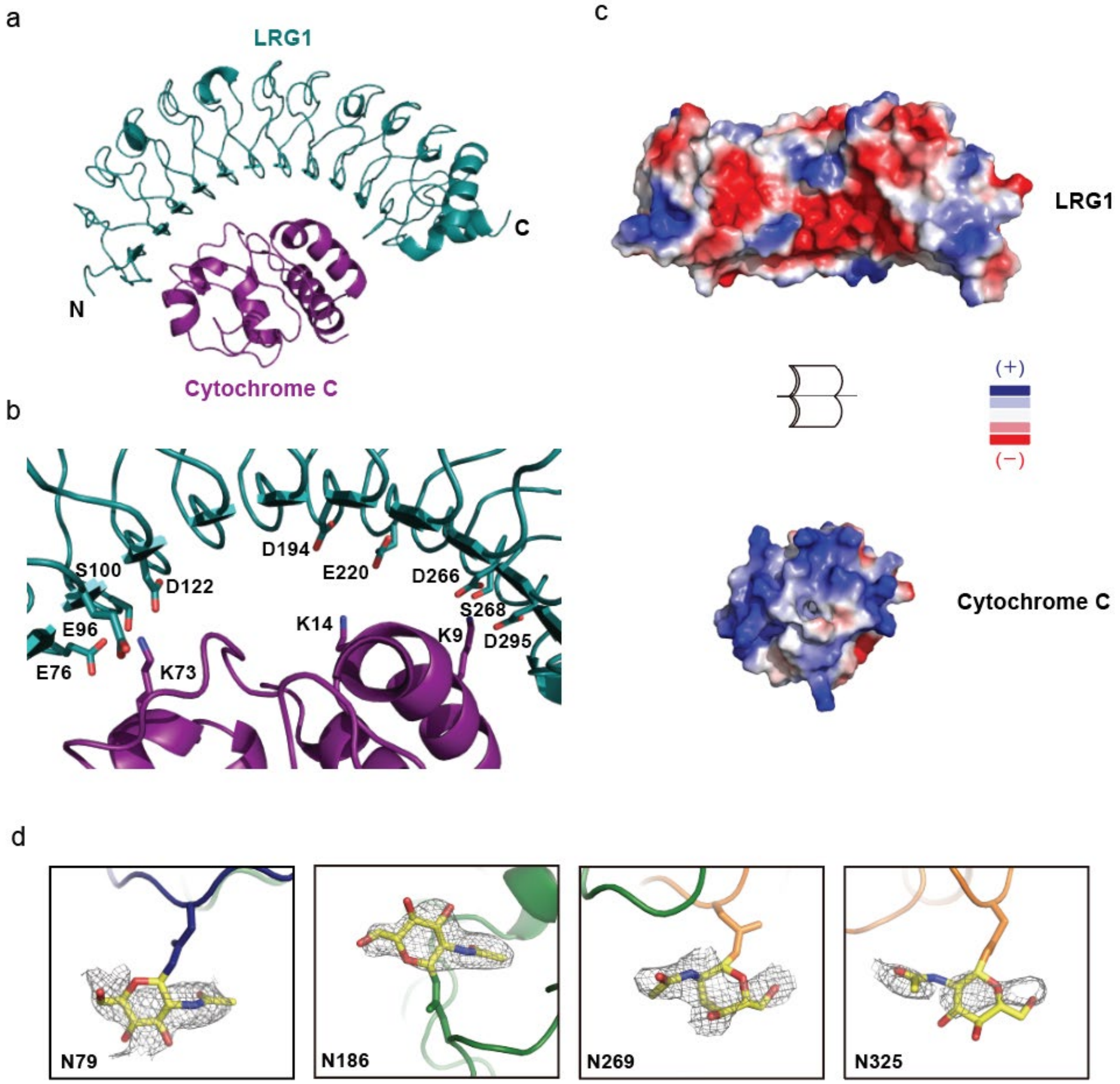

Supplementary Fig. 2 | Structure prediction of the human LRG1/cytochrome C complex

a Predicted structure of the human LRG1/cytochrome C complex using AlphaFold-multimer. LRG1 and cytochrome C are coloured teal and purple, respectively.

b Potential interactions between LRG1 and cytochrome C in the predicted structure of the human LRG1/cytochrome C complex. Residues involved in the potential interaction are displayed as sticks and labelled.

c Electrostatic potential of LRG1 and cytochrome C calculated according to the Poisson-Boltzmann equation in PyMOL. The structures are shown in open-book views, and the orientation of the LRG1 surface is identical to that in **Fig. 1c (left)**. Blue and red represent positively and negatively charged residues, respectively.

d The unbiased *m*Fo-*D*Fc Polder OMIT electron density maps for glycans were calculated by PHENIX. Maps are contoured at level of 3.5 σ for N79, 4.0 σ for N186, 4.0 σ for N269, and 4.0 σ for N325, respectively. N-acetylglucosamines (yellow) and asparagine residues are shown as sticks.

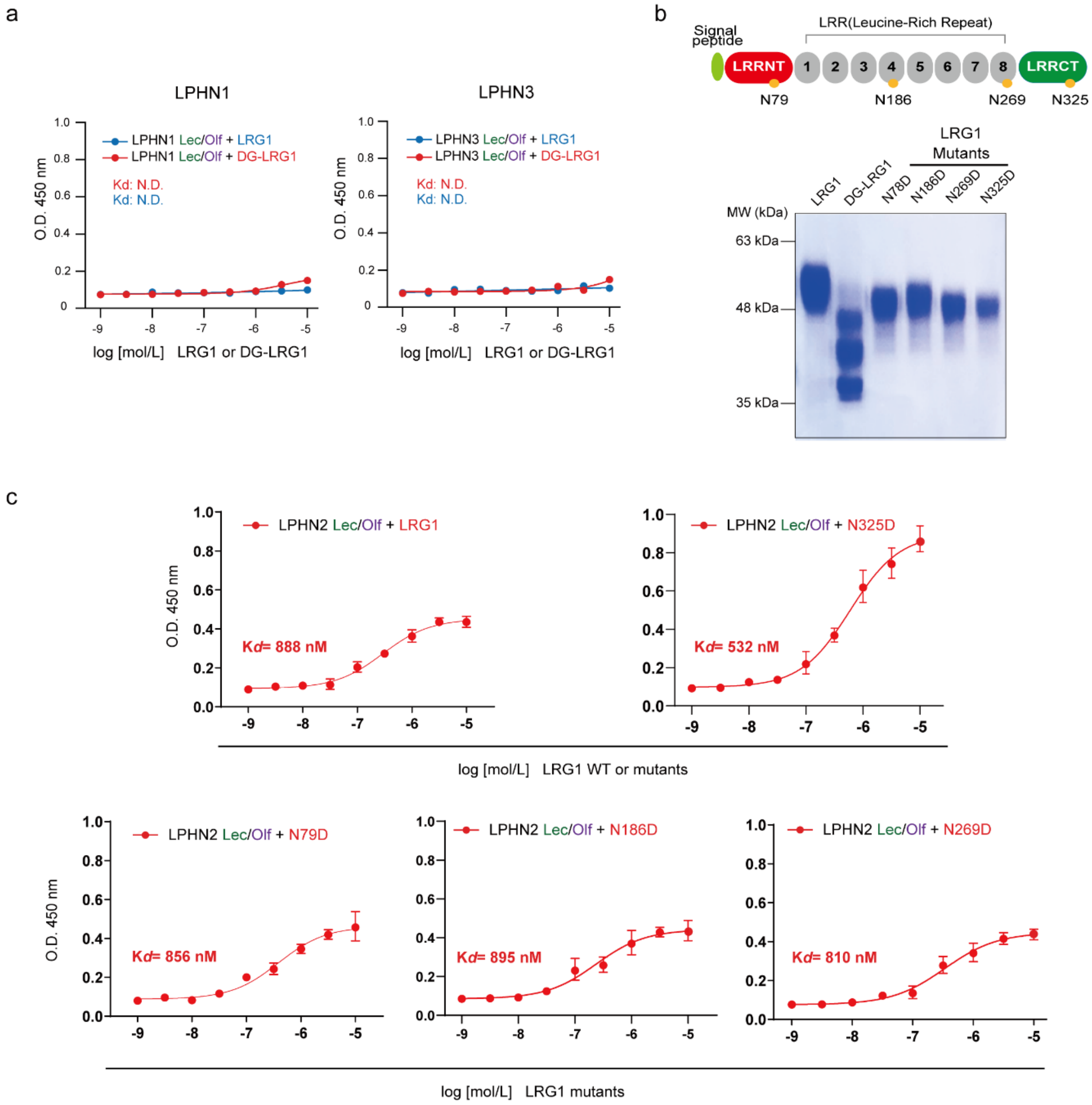

Supplementary Fig. 3 | Biochemical analysis of LRG1 and LPHNs

a Solid-phase binding assay assessing native LRG1 and DG-LRG1 binding to the Lec-Olf domains of LPHN1 and LPHN3. Lec-Olf domains of LPHN1 and LPHN3 (100 nM) were coated on a plate. After washing and blocking, different amounts (0.001, 0.005, 0.01, 0.05, 0.1, 0.5, 1, 5, and 10 µM) of native LRG1 or DG-LRG1 were added. We detected LRG1 or DG-LRG1 bound to coated proteins by ELISA using an anti-LRG1 antibody and anti-mouse secondary antibody.

b Schematic representation of the human LRG1 protein and highlighted glycosylation sites (N78D, N186D, N269D, N325D) as a yellow circle (top). Purified LRG1 mutants (N269D, N325D, N269D/N325D) and control LRG1 (native and de-glycosylated form) were analysed by SDS-PAGE and Coomassie Blue staining (bottom).

c The binding affinity of LRG1 N-glycan mutants (N79D, N186D, N269D, and N325D) to the LPHN2 ectodomain (Lec+Olf) was determined by a solid-phase binding assay.

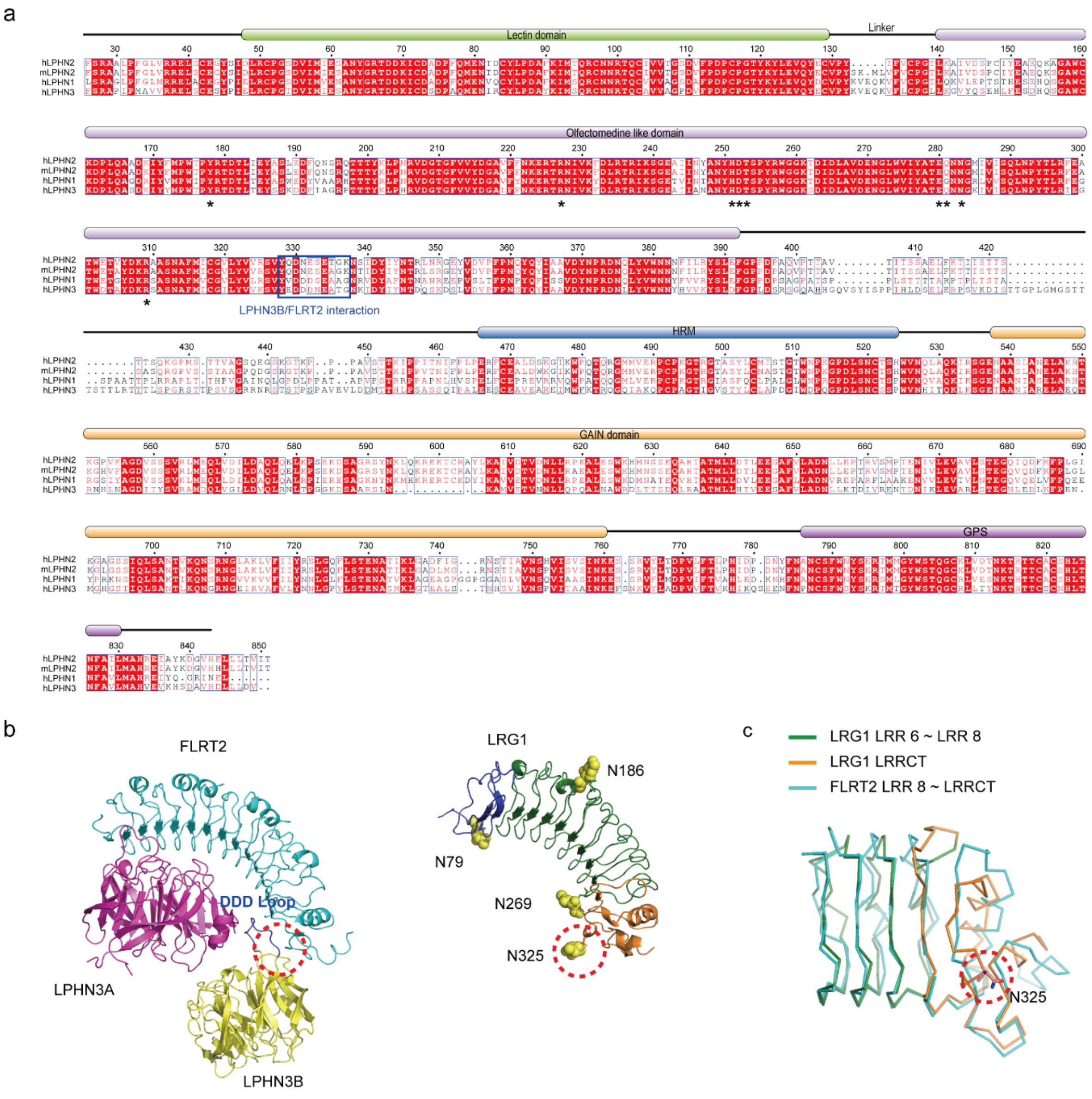

Supplementary Fig. 4 | Sequence alignment of LPHNs and structural comparison of LRG1 with FLRT2

a Sequence alignment of the ecto-domain of human LPHN1 (O94910.1), human LPHN2 (O95490.2), human LPHN3 (Q9HAR2.2), and mouse LPHN2 (Q8JZZ7). The sequence alignment was created using T-Coffee (http://tcoffee.crg.cat) and ESPript servers (http://espript.ibcp.fr). Lectin and Olf domains are followed by a hormone-binding region (HRM) adjacent to a GAIN domain that encompasses a cleavage site (GPS). The critical loop of LPHN3B for interaction with FLRT2 LRRCT is indicated as a blue box, and the key residues of LPHN2A for interacting with the N-terminal concave surface of FLRT2 are indicated by asterisks.

b Crystal structure of the FLRT2/LPHN3 complex (PDB ID: 5FTT) and LRG1. FLRT2, LPHN3A, and LPHN3B are coloured in cyan, purple, and yellow, respectively. The colour scheme for LRG1 (LRRNT, 8 LRR motifs, and LRRCT) is identical to that in **Fig. 1a**. The glycan on LRG1 (yellow sphere) and DDD loop in LPHN3B

involved in FLRT2 interactions are indicated. The critical glycosylation site for LRG1 function, N325, and the corresponding region in FLRT2 are marked with a red circle.

c Ribbon diagram comparing the structure of LRG1 to that of FLRT2 (PDB ID: 5FTT). Because LRG1 and FLRT2 contain 8 and 10 LRR motifs, respectively, we compared the LRR6–LRRCT domain of LRG1 with the LRR8–LRRCT domain of FLRT2 by superimposing their Cα chains. The critical glycosylation site, N325, for LRG1 function is indicated by a red circle.

Supplementary Table 1. Data collection and refinement statistics

	Human LRG1
**Data collection**	
Space group	$P6_322$
Cell dimensions	
a, *b*, *c* (Å)	143.02, 143.02, 113.73
α, β, γ (°)	90, 90, 120
Resolution (Å)	20-2.50 (2.59-2.50)[a]
R_{sym} or R_{merge} (%)[b]	14.6 (60.8)
$I/\sigma I$	46.4 (10.3)
Completeness (%)	100.0 (100.0)
Redundancy	36.6 (35.3)
**Refinement**	
Resolution (Å)	20.0-2.50
No. reflections	24,066
R_{work}/R_{free} (%)[c]	18.58/21.94
Average *B*-factor (Å^2)	38.57
R.M.S. deviations	
Bond length (Å)	0.01
Bond angle (°)	1.24
Ramachandran favoured (%)	92.51%
Ramachandran outliers (%)	0.00%
PDB entry	8H24

[a]Values in parentheses refer to the highest resolution shell.

[b]$R_{merge} = \sum_{hkl}\sum_i | I_{hkli} - \langle I_{hkli} \rangle | / \sum_{hkl}\sum_i \langle I_{hkli} \rangle$

[c]$R_{cryst} = \sum_{hkl}||F_o|-|F_c||/\sum|F_o|$

Supplementary Table 2. LRG1 genes for Consurf analysis

	LRG1 genes	
**Accession Numbers**	AAH34389.1 (*Homo sapiens*)	XP_032250335.1 (*Phoca vitulina*)
	XP_003819331.1 (*Pan paniscus*)	XP_035971916.1 (*Halichoerus grypus*)
	XP_001139381.2 (*Pan troglodytes*)	XP_027982170.1 (*Eumetopias jubatus*)
	XP_004059828.1 (*Gorilla gorilla gorilla*)	XP_025717182.1 (*Callorhinus ursinus*)
	XP_032033565.1 (*Hylobates moloch*)	KAI5931285.1 (*Manis javanica*)
	XP_002828525.1 (*Pongo abelii*)	XP_024899395.1 (*Pteropus alecto*)
	XP_003280613.1 (*Nomascus leucogenys*)	XP_006745640.1 (*Leptonychotes weddellii*)
	XP_003914749.2 (*Papio anubis*)	XP_012624713.1 (*Microcebus murinus*)
	EHH29497.1 (*Macaca mulatta*)	XP_037360138.1 (*Talpa occidentalis*)
	XP_011822754.1 (*Mandrillus leucophaeus*)	XP_022381508.1 (*Enhydra lutris kenyoni*)
	XP_033053196.1 (*Trachypithecus francoisi*)	XP_032989207.1 (*Rhinolophus ferrumequinum*)
	XP_011928516.1 (*Cercocebus atys*)	XP_021561072.1 (*Neomonachus schauinslandi*)
	XP_011798858.1 (*Colobus angolensis palliatus*)	XP_039734338.1 (*Pteropus giganteus*)
	XP_017702740.1 (*Rhinopithecus bieti*)	XP_034852503.1 (*Mirounga leonina*)
	XP_007993004.2 (*Chlorocebus sabaeus*)	KAI5758117.1 (*Gulo gulo luscus*)
	XP_012291861.1 (*Aotus nancymaae*)	XP_046542164.1 (*Equus quagga*)
	XP_017702741.1 (*Rhinopithecus bieti*)	XP_014698604.1 (*Equus asinus*)
	XP_002761694.2 (*Callithrix jacchus*)	XP_004277275.1 (*Orcinus orca*)
	XP_017383898.1 (*Cebus imitator*)	XP_032484646.1 (*Phocoena sinus*)
	XP_032108089.1 (*Sapajus apella*)	NP_001039642.1 (*Bos taurus*)
	XP_045401208.1 (*Lemur catta*)	XP_036740073.1 (*Manis pentadactyla*)
	XP_003788809.1 (*Otolemur garnettii*)	XP_024601807.1 (*Neophocaena asiaeorientalis*)
	XP_012624712.1 (*Microcebus murinus*)	XP_029774913.1 (*Suricata suricatta*)
	KAF6480955.1 (*Molossus molossus*)	ELR56248.1 (*Bos mutus*)
	XP_027440639.1 (*Zalophus californianus*)	XP_022412684.1 (*Delphinapterus leucas*)

Supplementary Table 3. Physiological and metabolic parameters of STZ-induced diabetic mice

2 weeks after treatment with PBS, DG-LRG1, N325D				
		STZ-induced diabetic mice		
	Normal	PBS	DG-LRG1	N325D
Body weight (g)	25.5±0.4	21.7±0.3*	21.6±0.2*	21.2±0.2*
Fasting glucose (mg/dl)	106.2±8.6	456.4±20.9*	460.4±14.9*	467.4±18.1*
Postprandial glucose (mg/dl)	139.4±15.1	576.2±11.1*	577.0±10.6*	570.8±11.7*
MSBP (mmHg)	130.9±2.0	129.4±2.4	133.1±2.3	125.9±2.1

Values are the mean ± SEM for n = 5 animals per group. *P < 0.05 vs. Normal group.
STZ, streptozotocin; MSBP, mean systolic blood pressure.

Supplementary Table 4. Antibody and Construct List

REAGENT or RESOURCE			SOURCE	IDENTIFIER
**Antibody**				
Rabbit polyclonal anti-LRG1			Sigma	Cat# HPA001888
Rabbit polyclonal anti-LPHN2			MyBioSource	MBS244156
Rabbit polyclonal anti-phospho-PI3 kinase p85 (Tyr458)/p55 (Tyr199)			Cell Signaling Technology	Cat# 4228
Rabbit polyclonal anti-PI3 kinase p85			Cell Signaling Technology	Cat# 4292
Rabbit polyclonal anti-phospho-Akt (Ser473)			Cell Signaling Technology	Cat# 9271
Rabbit polyclonal anti-Akt			Cell Signaling Technology	Cat# 9272
Rabbit polyclonal anti-phospho-NFkB p65 (Ser468)			Thermo Fisher Scientific	Cat# PA5-37721
Rabbit monoclonal anti-NF-κB p65 (D14E12)			Cell Signaling Technology	Cat# 8242
Rabbit polyclonal anti-βIII tubulin			Abcam	Cat# ab18207
Hamster monoclonal anti-PECAM-1			Millipore	Cat# MAB1398Z
Rabbit polyclonal anti-NG2			Millipore	Cat# AB5320
Goat anti-rabbit IgG (H+L) secondary antibody, HRP			Thermo Fisher Scientific	Cat# 31460
Goat anti-mouse IgG (H+L) secondary antibody, HRP			Thermo Fisher Scientific	Cat# 62-6520
Mouse monoclonal anti-β-actin			Santa Cruz Biotechnology	Cat# sc-47778
**Lentivirus**				
Control shRNA lentivirus particles			Santa Cruz Biotechnology	Cat# SC-108080
SMART vector mouse shRNA lentivirus of LPHN2			Dharmacon	Cat# V3SM7603-234963095
**Plasmid construct**	**Amino acid**	**Vector (company)**	**Cloning site**	**Description & reference**
Human LRG1	36-347	pcDNA3.1 (Invitrogen)	BamHI / XhoI	Crystallization
Human LRG1-Fc tag	36-577	pcDNA3.1 (Invitrogen)	BamHI / NotI	
Human LPHN2 lectin domain	26-95	pcDNA3.1 (Invitrogen)	BamHI / XbaI	
Human LPHN2 olfectamine domain	135-394	pcDNA3.1 (Invitrogen)	BamHI / XbaI	
Human LPHN2 lectin+olfectamine domain	26-394	pcDNA3.1 (Invitrogen)	BamHI / XbaI	
Human LPHN2 ecto full	26-796	pcDNA3.1 (Invitrogen)	BamHI / NotI	

Human LPHN1 lectin+olfectamine domain		pcDNA3.1 (Invitrogen)	BamHI / NotI	
Human LPHN3 lectin+olfectamine domain		pcDNA3.1 (Invitrogen)	BamHI / NotI	
Human LRG1(N79D)-Fc tag	36-577	pcDNA3.1 (Invitrogen)	BamHI / NotI	
Human LRG1(N186D)-Fc tag	36-577	pcDNA3.1 (Invitrogen)	BamHI / NotI	
Human LRG1(N269D)-Fc tag	36-577	pcDNA3.1 (Invitrogen)	BamHI / NotI	
Human LRG1(N325D)-Fc tag	36-577	pcDNA3.1 (Invitrogen)	BamHI / NotI	
PNGase F-GST	41-347	pcDNA3.1 (Invitrogen)	BamHI / EcoRI	
